# Microbiological Quality of Spanish Aged Cheeses and the Antimicrobial Resistance Profiles of Associated Enterococci, Staphylococci and *Enterobacterales*

**DOI:** 10.3390/foods15040721

**Published:** 2026-02-15

**Authors:** Celia Arraiz-Fernandez, Alba Martinez-Laorden, Gonzalo Ibañez-Torija, Elena Gonzalez-Fandos

**Affiliations:** Department of Food Technology, CIVA Research Center, University of La Rioja, Madre de Dios, 26006 Logrono, Spain

**Keywords:** food safety, antimicrobial resistance, cheese, dairy products, *E. coli*, enterococci, *S. aureus*

## Abstract

The aim of this work was to evaluate the microbiological quality of Spanish aged cheeses and the antimicrobial resistance of enterococci, staphylococci and *Enterobacterales*. A total of 60 aged cheeses produced in northern Spain were collected at the retail level. Mesophiles, lactic acid bacteria, staphylococci, enterococci, *Enterobacterales*, and yeast counts were determined. More microbial diversity was found in raw milk cheeses than in those elaborated with pasteurized milk. In general, lactic acid bacteria were the dominant microorganism, mainly *Lactiplantibacillus lactis*. High resistance rates were observed in *E. faecalis* strains isolated from raw milk cheeses (74.42%), being 9.30% multi-resistant. The dominant staphylococci found was *Staphylococcus equorum*. Multi-resistant *S. equorum* strains were isolated both from raw (1.69%) and pasteurized milk cheeses (9.09%). *Hafnia alvei* was the predominant bacterium in raw milk cheeses. Fifteen *Enterobacterales* strains, isolated from raw milk cheeses, showed multi-resistance (37.5%), and seven strains were ESBL producers (17.5%). *Escherichia coli* was only isolated from 5. 56% of raw milk cheeses, but all of them were extended-espectrum B-lactamase (ESBL) producers. The dominant yeast was *D. hansenni*, followed by *Kluyveromyces lactis*.

## 1. Introduction

Aged cheeses are formed by a complex microbial ecosystem in which bacteria, yeasts and moulds interact. These microorganisms play an essential role in the maturation of the product and its sensory quality [1]. The combination of microorganisms present in cheese is influenced by various factors, such as the heat treatment of milk (raw or pasteurized), the animal species of origin (goat, sheep, cow or mixtures of them), the technological production practices and the maturation conditions [2,3]. Studies conducted in several locations have revealed a high microbial diversity in cheeses, whose native microbiota plays a decisive role in defining both their organoleptic characteristics and their hygienic–sanitary quality [4,5,6].

Among the most relevant bacterial genera present in aged cheeses are *Lactiplantibacillus*, *Leuconostoc*, *Enterococcus* and *Staphylococcus*, which can have both a positive and negative influence on the quality and safety of the product [7,8]. Cheeses can contain lactic acid bacteria with technological and probiotic potential [8], but also undesirable or even pathogenic microorganisms, such as *Staphylococcus aureus* and *Escherichia coli*, whose presence has been described in various studies [9,10,11]. In particular, *Enterobacterales* and other bacterial groups contribute to both fermentation processes and food safety risks [7].

Aged raw milk cheeses are very popular and valued in Spain for their more complex and intense flavor compared to those elaborated with pasteurized milk. The microbial diversity in aged raw milk cheeses is more pronounced than in those made from pasteurized milk, as there is no prior heat treatment to reduce the initial load of microorganisms in milk [12].

In recent years, one aspect of growing concern is the presence of antibiotic-resistant bacteria in food, including milk products. Although there are some studies on antimicrobial-resistant bacteria in cheese, most of the studies have focused on fresh cheese [13,14,15,16], with the information on aged cheese being very limited [17]. Antibiotic-resistant bacteria can act as reservoirs of resistance genes with horizontal transfer capacity, assuming a potential risk to public health and food safety [18]. Furthermore, recent studies highlight that production chain management can influence the prevalence of resistant bacteria, highlighting the need for more integrated and sustainable control strategies [19].

In this context, it is interesting to study the microbiota present in aged cheeses from Spain, made with different types of milk and methods, with the dual objective of characterizing microbial diversity and evaluating the antibiotic resistance profile of the isolated bacteria. This knowledge is essential not only to better understand the microbiota of these products but also to assess their food safety and their role in the spread of antimicrobial resistance [8].

The aim of this study was to evaluate the microbiological quality of aged cheeses produced in northern Spain, to determine the prevalence of enterococci, staphylococci and *Enterobacterales*, and to characterize their antimicrobial resistance profiles.

## 2. Materials and Methods

### 2.1. Sampling and Microbiological Evaluation

A total of 60 samples of aged cheeses produced in three neighboring regions in northern Spain (Navarra, País Vasco and La Rioja) were collected between September and November 2024. The sampling strategy was designed in two stages. First, a preliminary survey was conducted to identify the types of aged cheeses produced in the three regions and to characterize the regional cheese production. Based on this information, a purposive sampling approach was implemented to ensure representation of the diversity of aged cheeses available, including both industrial and artisanal production systems. The main criterion for selecting the cheeses was that they were labelled as “aged” according to Spanish cheese standards, which establishes a minimum ripening period of 45 days for cheeses weighing < 1.5 kg and 105 days for cheeses weighing > 1.5 kg. No exclusion criteria were applied regarding producer size, production scale (artisanal vs. industrial), price range, or brand.

Of the 60 cheeses sampled, fifty-four (90%) were made from raw milk from different animals: sheep (41 cheeses), goat (6), cow (3) and milk mixtures (4). The other 6 samples (10%) were made from pasteurized milk from sheep (1), goat (4) and cow (1). It was ensured that the samples complied with preservation standards, maintaining a temperature of 4 °C from the time of purchase until arrival at the laboratory and subsequent analysis.

For microbiological determinations, 25 g of the cheese core was homogenized using sterile peptone water (0.1% *w*/*v*) (Oxoid, Basingstoke, Hampshire, UK) and a stomacher (IUL Instruments, Barcelona, Spain); each sample was analyzed in duplicate. The following determinations were performed: mesophiles, lactic acid bacteria, staphylococci, *Staphylococcus aureus*, enterococci, *Enterobacterales* and yeasts, as previously described [20,21]. To determine methicillin-resistant *Staphylococcus aureus* (MRSA) and vancomycin-resistant *Enterococcus* spp. (VRE), Brilliance MRSA Agar and Brilliance VRE Agar (Oxoid) were used respectively, as previously described by Gonzalez-Fandos and da Silva-Guedes [22]. The medium, temperature and incubation time for each microbial group are shown in Table 1.

### 2.2. Isolation and Identification of Strains

Three to six colonies were randomly selected from each duplicate sample and culture medium. The appearance of suspected colonies was considered when selective media were employed. Strains were purified using tryptic soy agar (Scharlau, Barcelona, Spain) and brain–heart infusion broth (Scharlau). The purified strains were stored at −80 °C. MALDI-TOF^®^ Biotyper model Microflex with the software FlexControl 3.4 and MBT Compass Version 4.180 (Bruker, Daltonik, Bremen, Germany) was used for bacterial identification. The reference database used was MBT Compass Explorer 4.1, which includes 10,833 reference profiles (Bruker). This equipment is intended for the characterization of bacteria by matrix assisted laser desorption–ionization (MALDI) time-of-flight mass spectrometry (TOF/MS). Hence, this technology identifies bacteria based on the mass spectra of cells or cellular components.

### 2.3. Phenotypic Antimicrobial Resistance of Enterococcus spp.

The antimicrobial susceptibility of *Enterococcus* spp. strains was tested against a total of 16 antimicrobials through the disc-diffusion technique on Mueller–Hinton agar (Oxoid). For each different medium and identification, one strain was selected. The following antibiotics discs were used: ampicillin (10 μg), chloramphenicol (30 μg), ciprofloxacin (5 μg), doxycycline (30 μg), enrofloxacin (5 μg), gentamicin (120 μg), imipenem (10 μg), levofloxacin (5 μg), linezolid (30 μg), minocycline (30 μg), nitrofurantoin (300 μg), norfloxacin (10 μg), teicoplanin (30 μg), tetracycline (30 μg), tigecycline (15 μg) and vancomycin (30 μg) (Oxoid). After incubation at 37 °C for 18 to 24 h, inhibition zones were measured and scored as resistant, susceptible or intermediate (reduced susceptibility) in accordance with the Clinical and Laboratory Standards Institute’s guidelines (CLSI) [23]. Strains were classified as multi-resistant (MDR) when they showed no susceptibility to three or more antimicrobial classes.

### 2.4. Phenotypic Antimicrobial Resistance of Staphylococcus spp. and Mammaliicoccus spp.

Phenotypic resistance determination assay was conducted on *Staphylococcus* spp. and *Mammaliicoccus* spp. strains following the guidelines of the CLSI using the disc diffusion technique [23]. Twenty-four antibiotics were evaluated: amikacin (30 μg), cefoxitin (30 μg), ceftaroline (30 μg), chloramphenicol (30 μg), ciprofloxacin (5 μg), clindamycin (2 μg), enrofloxacin (5 μg), erythromycin (15 μg), fusidic acid (10 μg), gentamicin (10 μg), kanamycin (30 μg), lincomycin (15 μg), linezolid (30 μg), mupirocin (200 μg), nitrofurantoin (300 μg), penicillin (10 μg), rifampicin (5 μg), streptomycin (10 μg), sulfadiazine (300 μg), tedizolid (2 μg), tetracycline (30 μg), tobramycin (10 μg), trimethoprim (5 μg) and vancomycin (30 μg) (Oxoid). Strains were classified as multi-resistant (MDR) when they showed no susceptibility to three or more antimicrobial classes. For the detection of methicillin-resistant *Staphylococcus* spp. and *Mammaliicococcus* spp. strains, the disc diffusion method in agar with cefoxitin was used as a fundamental tool in the detection of this resistance [23].

### 2.5. Phenotypic Antimicrobial Resistance of Enterobacterales

For the phenotypic analysis of *Enterobacterales*, the following antibiotics were tested for bacterial susceptibility: amikacin (30 µg), amoxicillin–clavulanic acid (20/10 µg), ampicillin (10 µg), ampicillin–sulbactam (10/10 µg), aztreonam (30 µg), cefepime (30 µg), cefotaxime (30 µg), cefoxitin (30 µg), cefpodoxime (10 µg), ceftazidime (30 µg), ceftriaxone (30 µg), chloramphenicol (30 µg), ciprofloxacin (5 µg), doripenem (10 µg), doxycycline (30 µg), enrofloxacin (5 µg), ertapenem (10 µg), gatifloxacin (5 µg), gentamicin (10 µg), imipenem (10 µg), kanamycin (30 µg), levofloxacin (5 µg), meropenem (10 µg), minocycline (30 µg), nalidixic acid (30 µg), nitrofurantoin (300 µg), norfloxacin (10 µg), piperacillin (100 µg), streptomycin (10 µg), sulfadiazine (300 µg), tetracycline (30 µg), tigecycline (15 µg), tobramycin (10 µg), trimethoprim (5 µg) and trimethoprim–sulfamethoxazole (1.25/23.75 µg). Phenotypic confirmation of extended-espectrum B-lactamase (ESBL) *Enterobacterales* producers was conducted in accordance with the CLSI [23].

### 2.6. Statistical Analysis

Analysis of variance was carried out using SPSS version 26 software (IBM SPSS Statistics). Tukey’s test for comparison of means was performed using the same program. The significance level was accepted at *p* < 0.05. Plate count data were converted to logarithms prior to their statistical analyses. All data were expressed as means ± standard deviation (SD). Statistical significance for the differences between raw and pasteurized milk cheeses, as well as among different animal milk species cheeses, was evaluated.

## 3. Results

### 3.1. Mesophiles

Mesophile counts in pasteurized (P) and raw milk cheeses (R) were 6.82 ± 1.09 and 6.82 ± 1.20, respectively (Supplementary Data, Table S1). No significant differences (*p* > 0.05) in mesophile counts were found between cheeses made from raw milk and those made from pasteurized milk (Supplementary Data, Table S1). Significant differences (*p* < 0.05) in mesophile counts were found between cheeses made from raw sheep or goat milk and those made from raw milk mixtures (Supplementary Data, Table S2).

The microorganisms identified from Plate Count Agar in cheese samples made from pasteurized and raw milk from different animal species are shown in Table 2 and Table 3, respectively. The microbial groups identified in pasteurized milk cheese samples were mainly lactic acid bacteria in those cheeses made from cow and goat milk. However, in cheese made from pasteurized sheep milk, the dominant microorganism was yeasts. Also, *Staphyloocooccaceae* and *Micrococcaceae* were isolated from cheese made with pasteurized goat or sheep milk.

More microbial diversity was found in raw milk cheeses than in those elaborated with pasteurized milk. Lactic acid bacteria were the dominant microorganism; however, *Staphyloocooccaceae*, *Micrococcaceae* and *Enterobacterales* were also found. Yeasts were also isolated in cheese made from raw sheep milk.

Other species identified included *Psychrobacter alimentarius* and *Acinetobacter schiindleri* in cheeses made from raw goat and sheep milk and *Bacillus cereus thuringiensis*, *Brochothrix thermosphacta*, *Microbacterium lacticum* and *Microbacterium paraoxydans* in cheeses made from raw sheep milk.

### 3.2. Lactic Acid Bacteria

Lactic acid bacteria counts in pasteurized milk cheeses (P) and raw milk cheeses (R) were 6.83 ± 1.67 and 7.39 ± 1.11, respectively (Supplementary Data, Table S1). No significant differences (*p* > 0.05) in lactic acid bacteria counts were found between pasteurized and raw milk cheeses (Supplementary Data, Table S1). No significant differences (*p* > 0.05) in mesophile counts were found among raw milk cheeses made from different animal species (Supplementary Data, Table S2).

The microorganisms identified from MRS media in cheese samples made from pasteurized and raw milk from different animal species are shown in Table 4. *Lactococcus lactis* was the dominant species in pasteurized milk cheeses, except in sheep milk cheeses, in which the dominant bacterium was *Lacticaseibacillus paracasei*. Other species, such as *Leuconostoc mesenteroides*, *Pediococcus pentosaceus* and *Enterococcus faecalis*, were also isolated.

More lactic acid bacteria diversity was found in raw milk cheeses than in pasteurized milk cheeses. The dominant species in raw milk cheeses was *Lacticaseibacillus paracasei*, except in those elaborated with a mixture of milks, in which the dominant bacterium was *Lactoococcus lactis*. Fourteen different lactic acid bacteria species were found in raw sheep milk cheeses: *Lacticaseibacillus paracasei*, *Lactiplantibacillus plantarum*, *Loigolactobacillus coryniformis*, *Lactococcus lactis*, *Lacticaseibacillus rhamnosus*, *Levilactobacillus brevis*, *Lentilactobacillus diolivorans*, *Leuconostoc mesenteroides*, *Pediococcus pentosaceus*, *Latilactobacillus curvatus*, *Lactiplantibacillus paraplantarum*, *Latilactobacillus sakei*, *Enterococcus faecalis* and *Enterococcus faecium*.

Five different lactic acid bacteria species were isolated from raw goat milk cheeses (*Lacticaseibacillus paracasei*, *Lactiplantibacillus plantarum*, *Lactococcus lactis*, *Leuconostoc pseudomesenteroides* and *Leuconostoc mesenteroides*).

Four different lactic acid bacteria species were isolated from raw cow milk cheeses (*Lacticaseibacillus paracasei*, *Lactiplantibacillus plantarum*, *Lactococcus lactis* and *Leuconostoc pseudomesenteroides*).

### 3.3. Enterococci

Enterococci counts below the detection limit (2 log CFU/g) were found in three of six pasteurized milk cheeses (50%). The other three samples, made from raw goat milk, showed counts between 4.01 and 6.05 log CFU/g with an average number of 4.94 ± 0.84 (Supplementary Data, Table S1).

Enterococci counts below 2 log CFU/g were found in 8 of 54 raw milk cheeses (14.8%). The other 46 samples showed counts between 2.24 and 7.23 log CFU/g with an average number of 5.24 ± 1.33 log CFU/g (Supplementary Data, Table S1). Significant differences (*p* < 0.05) in enterococci counts were found between raw and pasteurized milk cheeses (Supplementary Data, Table S1). Significant differences (*p* < 0.05) in enterococci counts were found between raw sheep milk cheese and raw cow milk cheeses, as well as between raw sheep, goat or cow milk cheeses and raw milk mixture cheeses (Supplementary Data, Table S2).

Table 5 shows the species identified from Bile Esculin Azide agar. *Enterococcus faecalis* was the dominant enterococci in pasteurized goat milk cheeses. *E. durans* was the dominant species in raw goat and cow milk cheeses, while in raw sheep milk cheeses *E faecalis* was the dominant species. When using Brilliance VRE agar, *E. gallinarum* was found in five raw milk cheeses.

**Figure 1:**
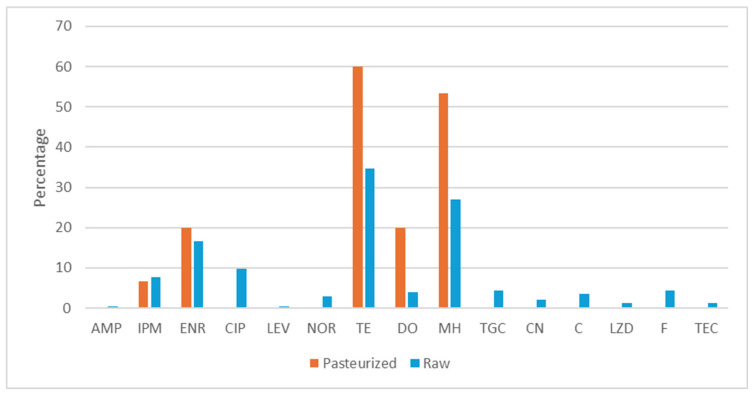
Percentage of enterococci resistance to antibiotics isolated from aged cheeses elaborated with pasteurized or raw milk. AMP: ampicillin, IMP: imipenem, ENR: enrofloxacin, CIP: ciprofloxacin, LEV: levofloxacin, NOR: norfloxacin, TE: tetracycline, DO: doxycycline, MH: minocycline, TGC: tigecycline, CN: gentamicin, C: chloramphenicol, F: nitrofurantoin, LZD: linezolid, TEC: teicoplanin.

The antimicrobial resistance phenotype of 15 enterococci strains isolated from pasteurized milk cheeses (10 strains of *E. faecalis*, 4 strains of *E. faecium* and 1 strain of *E. maladoratus*) and 248 strains isolated from raw milk cheeses (86 strains of *E. faecalis*, 71 strains of *E. faecium*, 46 strains of *E. durans*, 21 strains of *E. gilvus*, 5 strains of *E. gallinarum*, 15 strains of *E. hirae* and 4 strains of *E. malodoratus*) was evaluated (Figure 1). None of the strains showed resistance to vancomycin, but strains resistant to the other 15 antibiotics tested were detected.

Regarding the enterococci isolated from pasteurized milk cheeses, the highest resistance rates were observed against tetracycline (60%); 40% of the strains were susceptible to all the antibiotics evaluated (one strain of *E. malodoratus*, four strains of *E. faecalis* and one strain of *E. faecium*). Only one strain of *E. faecium* showed multi-resistance (6.67%, one of fifteen).

The highest resistance rates of enterococci isolated from raw milk cheeses were observed against tetracycline (34.68%), minocycline (27.02%) and enrofloxacin (16.53%). Thirteen strains were multi-resistant (5.24%), 12 of them were isolated from raw sheep milk cheeses and one from raw mixture milk cheese.

Overall, *E. faecalis*, had the greatest resistance rates (74.42%), followed by *E. gallinarum*, *E. faecium*, *E. gilvus* and *E. hirae* (60%, 49.3%, 39.13%, 23.83% and 20%, respectively). The higher multi-resistant rates were observed in *E. faecalis* (9.30%), followed by *E*. *gilvus*, *E. durans* and *E. faecium* (4.76%, 4.08% and 2.82%, respectively).

### 3.4. Staphylococci

Staphylococci counts were observed in all the pasteurized milk cheeses, with counts between 2.99 and 6.01 log CFU/g and an average number of 3.94 ± 1.01 log CFU/g. Staphylococci counts below 2 log CFU/g were found in 24 of 54 raw milk cheeses (44.4%). The counts in the other 30 cheeses ranged between 2.30 and 6.53 log CFU/g, with an average of 4.01 ± 1.03 log CFU/g (Supplementary Data, Table S1). Significant differences (*p* < 0.05) in staphylococci counts were found between raw and pasteurized milk cheeses (Supplementary Data, Table S1). Significant differences (*p* < 0.05) in staphylococci counts were found between raw sheep milk cheeses and raw cow milk cheeses, as well as between raw sheep milk cheeses and raw milk goat cheeses. Also, significant differences (*p* < 0.05) were found between raw cow and goat milk cheeses and raw mixture milk cheeses (Supplementary Data, Table S2). The results observed in Baird–Parker agar showed that *S. aureus* counts were below 2 log CFU/g in all the samples.

Table 6 shows the species identified from Mannitol Salt Agar. The only staphylococci found in pasteurized milk cheeses was *Staphylococcus equorum*. This species was also dominant in all the raw milk cheeses. However, other species were found in raw cow milk cheeses (*S. epidermidis*) and in raw mixture of milk cheeses (*S. capitis*). Twelve different staphylococci species were identified in raw sheep milk cheeses (*S. equorum*, *S. simulans*, *S. saprophyticus*, *S. pasteuri*, *S. epidermidis*, *S. ureilyticus*, *S. haemolyticus*, *Mammaliicoccus sciuri*, *S. aureus*, *S. capitis*, *S. hominis* and *S. piscifermentans*). *S. aureus* was only isolated from one raw sheep cheese. No *S. aureus* was isolated from Brilliance MRSA agar.

**Figure 2:**
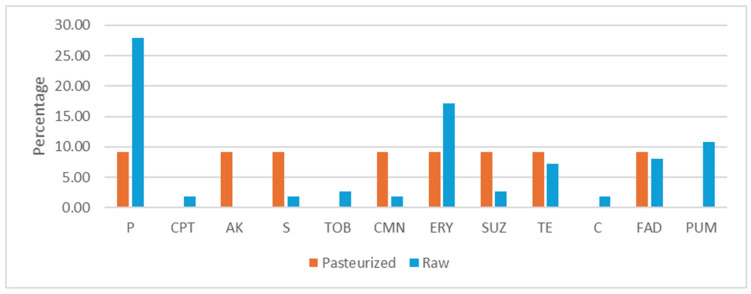
Percentage of staphylococci resistance to antibiotics isolated from aged cheeses elaborated with pasteurized or raw milk. P: penicillin, CPT: ceftaroline, AK: amikacin, S: streptomycin, TOB: tobramycin, CMN: clindamycin, ERY: erythromycin, SUZ: sulfadiazine, TE: tetracycline, C: chloramphenicol, FAD: fusidic acid, PUM: mupirocin.

The antimicrobial resistance phenotype of 11 strains of *S. equorum* isolated from pasteurized milk cheeses was evaluated (Figure 2). Ten strains were susceptible to all the antibiotics evaluated (90.91%) and one strain showed multi-resistance (9.09%). The multi-resistant strain was isolated from a pasteurized cow milk cheese and showed resistance to 8 antibiotics: penicillin, amikacin, streptomycin, clindamycin, erythromycin, sulfadiazine, tetracycline and fusidic acid.

The antimicrobial resistance phenotype of 111 strains of staphylococci isolated from raw milk cheeses was evaluated (Figure 2). The distribution of the strains was as follows: *S. equorum* (59 strains, 53.15%), *S. simulans* (14, 12.61%), *S. epidermidis* (12, 10.81%), *S. saprophyticus* (8, 7.21%), *S. hominis* (5 strains, 4.5%), *S. pasteuri* (3, 2.70%), *S. capitis* (2, 1.8%), *S. aureus* (2, 1.8%), *S. ureilyticus* (1, 0.90%), *S. haemolyticus* (1, 0.90%), *Mammaliicoccus sciuri* (1, 0.90%), *Mammaliicoccus vitulinus* (1, 0.90%) and *S. piscifermentans* (1, 0.90%). The highest resistance rates were observed against penicillin (27.93%) and erythromycin (17.12%). Eight strains showed multi-resistance (3 strains of *S. epidermidis*, 1 *S. equorum*, 2 *S. hominis* and 2 *S. saprophyticus*) (7.21%). The *S. aureus* strains isolated only showed resistance to penicillin, ceftaroline and erythromycin. Regarding *S. equorum* strains isolated from raw milk cheeses, 32.20% showed resistance to penicillin, 3.39% to tetracycline and 10.17% to erythromycin. Only one strain isolated from raw goat cheese (1.69%) was multi-resistant, showing resistance against tobramycin, chloramphenicol and mupirocin.

### 3.5. Enterobacterales

*Enterobacterales* were only detected in one cheese sample made from pasteurized goat milk (16.7%) with counts of 3.13 ± 0.00 log CFU/g (Supplementary Data, Table S1). In raw milk cheeses, *Enterobacterales* were found in 13% of cheeses (7/54) with counts ranging from 1.30 to 6.73 log CFU/g and average counts of 3.25 ±1.87. No significant differences (*p* > 0.05) in *Enterobacterales* counts were found between raw and pasteurized milk cheeses (Supplementary Data, Table S1). However, significant differences (*p* < 0.05) were found between raw sheep milk cheese and raw cow milk cheeses, as well as between raw sheep and goat milk cheeses. Also, significant differences (*p* < 0.05) were found between cow milk cheeses and raw mixture milk cheeses (Supplementary Data, Table S2).

Table 7 shows the species distribution. *Hafnia alvei* was the predominant bacterium in raw milk cheeses, while the predominant one in pasteurized cheeses was *Lelliotia ammigena. E. coli* was only isolated from three raw milk cheeses (5.56%), two from sheep milk and another from cow milk.

**Figure 3:**
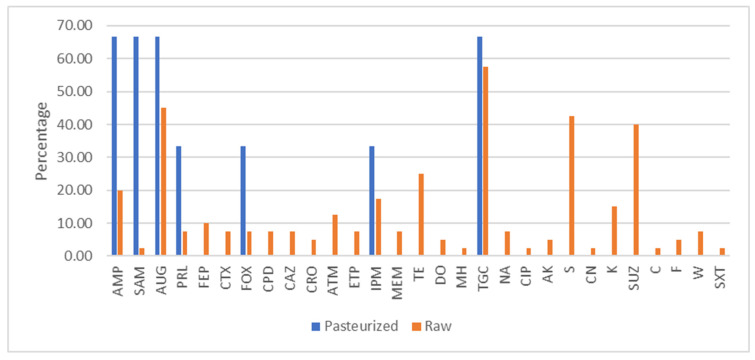
Percentage of *Enterobacterales* resistance to antibiotics isolated from aged cheeses elaborated with pasteurized or raw milk. AMP: ampicillin, SAM: ampicillin–sulbactam, AUG: amoxicillin–clavulanic acid, PRL: piperacillin, FEP: cefepime, CTX: cefotaxime, FOX: cefoxitin, CPD: cefpodoxime, CAZ: ceftazidime, CRO: ceftriaxone, ATM: aztreonam, ETP: ertapenem, IPM: imipenem, MEM: meropenem, TE: tetracycline, DO: doxycycline, MH: minocycline, TGC: tigecycline, NA: nalidixic acid, CIP: ciprofloxacin, AK: amikacin, S: streptomycin, CN: gentamicin, K: kanamycin, SUZ: sulfadiazine, C: chloramphenicol, F: nitrofurantoin, W: trimethoprim, SXT: trimethoprim–sulfamethoxazole.

The antimicrobial resistance phenotype of three strains of *Enterobacterales* isolated from pasteurized milk cheeses was evaluated (*Enterobacter bugandensis*, *Enterobacter hormaechei* and *Lelliottia amnigena*) (Figure 3). No strain showed multi-resistance. No strain was extended-espectrum B-lactamase (ESBL)-producing.

The antimicrobial resistance phenotype of 40 strains of *Enterobacterales* isolated from raw milk cheeses was evaluated (Figure 3). The distribution of the strains was as follows: *Hafnia alvei* (26 strains), *E. coli* (3 strains), *Klebsiella oxytoca* (4 strains), *Raoultella planticola* (3 strains), *Enterobacter kobei* (3 strains), *Leclercia adecarboxylata* (3 strains) and *Lelliottia amnigena* (3 strains).

The highest resistance rates were observed against tigecycline (57.50), sulfadiazine (40%), amoxicillin–clavulanic acid (45%) and streptomycin (42.5%). Fifteen strains showed multi-resistance (37.5%), and seven strains were ESBL producers (17.5%).

All the *E. coli* strains evaluated showed multi-resistance and were ESBL producers. *E. coli* isolates showed resistance against tetracycline (100%), streptomycin (100%), kanamycin (66.67%), trimethoprim (66.67%), ampicillin (66.7%), amoxicillin–clavulanic (66.7%), piperacillin (66.67%), chloramphenicol (33.33%), gentamicin (33.33%), sulfadiazine (33.33%) and trimethoprim–sulfamethoxazole (33.33%). However, no resistance was observed against carbapenems (ertapenem, meropenem, imipenem) or fluoroquinolones. One *E. coli* strain isolated from cow cheese showed resistance to 11 antibiotics.

Eleven strains of *Hafnia alvei* (42.31%) showed multi-resistance. The four strains of *Klebsiella oxytoca* isolated from cow cheese were not multi-resistant, but all of them were ESBL producers. All of them showed resistance to ampicillin and only one to streptomycin. One strain of *Lelliottia amnigena* isolated from sheep milk showed resistance to 13 antibiotics.

### 3.6. Yeasts

Yeast counts in pasteurized cheeses ranged between 3.30 and 6.27 hog CFU/g with a mean value of 4.62 ± 1.05. Yeast counts below 2 log CFU/g were found in 61.1% of the raw milk cheeses. In the other cheeses, yeast counts ranged between 2.45 and 5.43 with an average value of 3.96 ± 0.93 (Supplementary Data, Table S1).

The species identified are shown in Table 8. The dominant yeast was *D. hansenni*, followed by *Kluyveromyces lactis*. *Yarrowia lipolytica*, *Kluyveromyces marxianus*, *Pichia fermentans*, *Candida zeylanoides* and *Clavispora lusitaniae* were also isolated; however, to a lesser extent.

## 4. Discussion

### 4.1. Microbiota in Cheese

As in the present work, other authors have reported high lactic acid bacteria counts in aged cheese (7.75 ± 0.20 log CFU/g) [19]. Several studies have shown that lactic acid bacteria are the dominant bacteria in cheese; the most common genera found are: *Lactococcus*, *Lactiplantibacillus*, *Lacticaseibacillus* and *Enterococcus* [19]. We also found that lactic acid bacteria were the dominant microorganisms in cheese, except in those made from pasteurized sheep milk, in which the dominant microorganisms were yeasts. As previously reported, we found more microbial diversity in raw milk cheeses than in pasteurized milk cheeses [24,25]. Although lactic acid bacteria were the dominant microorganisms, *Staphylococcaceae* and *Enterobacterales* were also found.

In the present work, yeasts were also isolated in cheeses made from pasteurized and raw sheep milk. Other Gram-negative bacteria (*Psychrobacter alimentarius* and *Acinetobacter schiindleri* were found in cheese made from raw goat and sheep milk. Other Gram-positive bacteria, such as *Bacillus cereus thuringiensis*, *Brochothrix thermosphacta*, *Microbacterium lacticum* and *Microbacterium paraoxydans,* were also found in cheeses made from raw sheep milk. The genera *Staphylococcus*, *Psychrobacter*, *Acinetobacter* and *Bacillus* have also been detected in cheese by other authors [19,24,26].

Cheese is one of the most diverse of all foodstuffs, since it is produced following different technological processes and using milk from different animal species (goats, cows, sheep, buffaloes, yaks) [1]. The microorganisms present in cheese are affected by biotic and abiotic factors, thus different proportions of lactic acid bacteria and other microorganisms have been reported in cheese [1].

We observed that the dominant bacteria in cheese elaborated with pasteurized cow and goat milk was *Lc. lactis*, while in those elaborated with sheep milk, was *Lacticaseibacillus paracasei* (50%), followed by *Lc. lactis* (40%). The dominant species in cheeses elaborated with cow, goat and sheep raw milk was *Lacticaseibacillus paracasei*, while in those elaborated with a mixture of raw milk, was *Lc. lactis*. Other authors have also reported that *Lc. lactis* is the most abundant microorganism in cheeses [2,24]. In contrast, Santamarina-García et al. reported that in raw sheep-milk-aged cheeses from Navarra (Spain), the dominant lactic acid bacteria were *Lactiplantibacillus* species (20.8%), followed by *Lacticaseibacillus*, specifically *L. paracasei* (16.7%), while we found that *L. paracasei* was the dominant lactic acid bacteria (51.71%) [19]. Kamilari et al. also reported that lactic acid bacteria were the predominant bacteria in a Cyprus-aged cheese made from pasteurized goat and sheep milk, the most common genera detected being *Lactiplantibacillus*, *Leuconostoc*, *Pediococcus* and Weissella [26]. These authors analyzed 17 cheeses versus 60 analyzed in the current study [26].

We found more lactic acid bacteria diversity in raw milk cheeses than in pasteurized milk cheeses. The microbial populations present in cheeses are affected by the animal source of milk and pasteurization [24,25]. Ten different genera and 14 different lactic acid bacteria species were found in cheese made with sheep raw milk: *Lacticaseibacillus paracasei*, *Lactiplantibacillus plantarum*, *Loigolactobacillus coryniformis*, *Lc. lactis*, *Lacticaseibacillus rhamnosus*, *Levilactobacillus brevis*, *Lentilactobacillus diolivorans*, *Leuconostoc mesenteroides*, *Pediococcus pentosaceus*, *Latilactobacillus curvatus*, *Lactiplantibacillus paraplantarum*, *Latilactobacillus sakei*, *E. faecalis* and *E. faecium*. In cheese made from raw goat milk, only four genera and five species of lactic acid bacteria were isolated: *Lacticaseibacillus paracasei*, *L. plantarum*, *L. lactis*, *Leuconostoc pseudomesenteroides*, and *Leuconostoc mesenteroides*. Less diversity was found in cheese made from raw cow milk, only four different genera and four species: *Lacticaseibacillus paracasei*, *L. plantarum*, *Lc. lactis*, *Leuconostoc pseudomesenteroides*. The genera present in cheeses are affected by the animal source of milk and pasteurization [24]. Our results contrast with those reported by Quigley et al., who observed higher microbial diversity in 62 cow milk cheeses (21 different bacterial genera detected) than in goat milk cheeses (eight different bacterial genera), or sheep milk cheeses (two bacterial genera, *Lactococcus* and *Lactiplantibacillus*, detected) [24]. It should be pointed out that the technological processes also affect the microbiota present in cheese [2].

As in the present work, enterococci are often found in cheeses [24,27,28]. We observed that *E. faecalis* was the dominant enterococci in cheese made from pasteurized goat milk. In raw cow and goat cheeses, *E. durans* was the dominant species, while in raw sheep milk cheeses, *E. faecalis* was the dominant species. Some authors have reported that *E. faecalis* is the most often isolated enterococci from cheese [29]. In contrast, other authors have pointed out that *E. faecium* is the most common species isolated [27]. Also, *E. durans* has been isolated from cheeses by other authors [1,29,30]. Santamarina-García et al. also found *E. hirae* in raw sheep-milk-aged cheeses from Spain [19]. The enterococci species vary depending on the cheese type [28].

*Staphylococcus* spp. are often found in cheese [25]. As other authors have found that *Staphylococcus equorum* is the most abundant *Staphylococcus* in cheese [17,29,30]. A higher prevalence of *S. aureus* in 10 cheese samples has been reported by Erhardt et al. (10%) than in the present work (1.67%) [31].

In the present work, *Enterobacterales* were mainly represented by the genera *Hafnia*, *Enterobacter*, *Raoultella* and *Lelliottia*. *E. coli*, *Klebisella* and *Lecrercia* were isolated in lesser extent. In contrast, Ritschard et al. isolated mainly *Proteus* and *Morganella,* and to a lesser extent *Citrobacter*, *Enterobacter*, *Serratia*, *Providencia* and *Hafnia* from 15 Swiss cheeses [7].

*Hafnia alvei* is often isolated from cheese [32]. *Enterobacter bugandensis* and *Enterobacter hormaechei* have been previously reported in Brazilian and Italian cheeses, respectively [7,33]. *K. oxytoca* and *Raoultella ornithinolytica* have been previously found in cheese [7,32].

A higher prevalence of *E. coli* in cheeses (22.2–78.7%) has been reported by other authors than in the present work (5%) [9,30,33]. The presence of *E. coli* and other *Enterobacterales* in aged raw milk cheeses is considered an indicator of fecal contamination, mainly during milking, and could represent a significant public health risk [34].

Regarding yeast, Cenci-Goga et al. also detected yeast in a high percentage of the 69 cheeses analyzed (73.9%) [6]. As in the present work, other authors have observed that *Debaryomyces hansenii* was the most abundant yeast in cheese [4,6]. Also, *Kluyveromyces lactis*, *Kluyveromyces marximus*, *Yarrowia lipolytica* and *Pichia fermentans* have been previously reported in cheeses [4,6,35]. In French cheeses, *Clavispora lusitaniae* and other *Candida* spp. have also been isolated: *C. sake*, *C. ralunensis*, *C. parapsilosis*, *C. intermedia*, *C. incospicua*, *C. catenulate* and *C. atlantica*, but not *C. zeylanoides* [35]. Yeasts can contribute to flavor and appearance in cheese but can also cause spoilage or generate undesirable flavors [4]. *Debaryomyces hansenii* and *Kluyveromyces lactis* may contribute to flavor development in cheese, while *Yarrowia lipolytica* is the main yeast associated with spoilage in cheese [4,6].

### 4.2. Antimicrobial Resistance in Strains Isolated from Cheese

As other authors we have isolated resistant enterococci from cheese. Výrostková et al., after analyzing 20 cheese samples, reported that *E. faecalis* had the greatest resistance rates (74.42%), followed by *E. gallinarum*, *E. faecium*, *E. gilvus* and *E. hirae* (60%, 49.3%, 39.13%, 23.83% and 20%, respectively) [36]. These results are in line with those observed in the current work. In contrast, Santamarina-García et al. reported that *E. durans* and *E. faecium* showed the highest resistance rates (100% and 80.0%, respectively), while *E. hirae* and *E. faecalis* showed resistance rates of 57.1% and 45.4%, respectively [19]. Higher multi-resistant rates have been observed in sheep milk cheeses from Brazil (24.2%) and in goat and sheep milk cheeses from Slovakia (80%) than in the present work (5.24%) [28,30]. We did not find any enterococci resistant to vancomycin. However, other authors have reported high resistance rates to vancomycin in *E. durans* (66.7%) and *E. faecium* (100%) isolated from 83 cheese samples [36].

Also, Sri Prabakusuma et al. reported resistance to penicillin and erythromycin in *S. aureus* strains isolated from 124 cheeses [37]. Moreover, these authors found *S. aureus* strains resistant to cefoxitin and tetracycline and a high percentage of multi-resistant strains (34.78%). We found 7.21% staphylococci strains were multi-resistant, but none of them was *S. aureus*. Although other authors have reported that *S. equorum* strains isolated from cheese only showed moderate resistance to chloramphenicol and were susceptible to penicillin, streptomycin, clindamycin, erythromycin, and tetracycline, we found *S. equorum* strains resistant to these antibiotics [17]. Even more, we observed *S. equorum* strains resistant to amikacin, sulfadiazine, fusidic acid, tobramycin and mupirocin. The resistance of *S. equorum* found in the current study is relevant, since this *Staphylococcus* spp. is the most abundant one in cheese [1,17].

Other authors have also observed that *E. coli* strains isolated from cheese showed high resistance rates [10]. In the present study, *E. coli* isolates showed resistance against tetracycline (100%), streptomycin (100%), kanamycin (66.67%), trimethoprim (66.67%), ampicillin (66.7%), amoxicillin–clavulanic (66.7%), piperacillin (66.67%), chloramphenicol (33.33%), gentamicin (33.33%), sulfadiazine (33.33%) and trimethoprim–sulfamethoxazole (33.33%). Also, Hussein et al. reported high resistance levels of *E. coli* isolated from cow milk cheeses from Lebanon against ampicillin (67%), amoxicillin–clavulanic (51%), streptomycin (58%), tetracycline (40%), gentamicin (41%), kanamycin (36%), chloramphenicol (28%), and trimethoprim–sulfamethoxazole (38%) [10]. However, we did not observe any resistance against carbapenems (ertapenem, meropenem, imipenem) or fluoroquinolones, while Hussein et al. reported resistance against imipenem (19%), meropenem (37%), ciprofloxacin (5%) and norfloxacin (4%) [10]. These authors also reported resistance against cefepime (19%), while we did not detect any resistance against this antimicrobial [10]. As Praça et al. in aged raw milk cheeses from Portugal (96 samples analyzed), we found *E. coli* strains resistant to ampicillin, amoxicillin–clavulanic acid, chloramphenicol, tetracycline and trimethoprim [9]. Özadam and Özpina also found *K. oxytca* and *E. coli* strains ESBL producers when analyzing 83 cheese samples [32]. These authors isolated *H. alvei* ESBL producers from cheese [34]. In contrast, we did not isolate any *H. alvei* ESBL producer. The prevalence of beta-lactamase-producing *Enterobacterales* in food of animal origin is considered of special concern, since they could be a reservoir of ESBL producers for humans [38].

The high resistance rates against tetracyclines and ß-lactams are probably due to their frequent use in livestock. Besides the importance of hygienic measures during the milking stage, the presence of resistant bacteria in the farm environment (soil, water and wildlife) highlights the environmental reservoirs of resistance genes that could be transferred to milk [39]. This underlines the need to control both the dairy industry and the farm environment. Since resistance genes were not investigated in this study, further studies for molecular confirmation are required.

## 5. Conclusions

This study highlights that multi-resistant enterococci, staphylococci and *Enterobacterales* can be present in aged cheeses. Resistance genes were not investigated in this study, thus further studies for molecular confirmation are required. Since aged cheese could be a source of ESBL-producing *E. coli* and ESBL-producing *Klebsiella oxytoca*, special measures should be taken in the framework of the One Health approach. It is also essential to develop strategies for controlling the presence of resistant microorganisms in raw milk and the dairy farm environment, and along the manufacturing cheese process.

## Figures and Tables

**Table 1:** Media, temperature and incubation times used for microbiological determinations.

Microbial Group	Media (Manufacturer)	Temperature (°C)	Time (h)
Mesophiles	Plate Count Agar (Scharlau)	30	48
Lactic acid bacteria	De Man Rogosa Sharpe Agar (Scharlau) ^1^	30	72
Staphylococci	Mannitol Salt Agar (Oxoid)	35	36
*S. aureus*	Baird Parker Agar Base (Oxoid)	37	48
*Enterococcus* spp.	Bile Esculin Azide Agar (Scharlau)	37	48
*Enterobacterales*	Violet Red Bile Glucose Agar (Oxoid)	37	24
Yeasts	Potato Dextrose Agar (Scharlau)	25	48
Methicillin-resistant *S. aureus*	Brilliance MRSA Agar (Oxoid)	37	24
Vancomycin-resistant *Enterococcus* spp.	Brilliance VRE Agar (Oxoid)	37	24

^1^ Incubated under anaerobic conditions.

**Table 2:** Microorganisms identified in pasteurized milk cheeses from different animal species (cow, sheep and goat) isolated from Plate Count Agar.

Type of Milk	Microbial Group	Percentage (%)	Species	Percentage (%)
Cow	Lactic acid bacteria	100	*Lactococcus lactis*	100
Goat	Lactic acid bacteria	94.88	*Lactococcus lactis*	74.36
			*Leuconostoc mesenteroides*	2.56
			*Leuconostoc pseudomesenteroides*	17.95
	*Microccaceae*	2.56	*Micrococcus luteus*	2.56
	Yeasts	2.56	*Debaryomyces hansenii*	2.56
Sheep	Yeasts	77.78	*Debaryomyces hansenii*	77.78
	*Staphyloocooccaceae*	*22.22*	*Staphylococcus equorum*	22.22

**Table 3:** Microorganisms identified in raw milk cheeses from different animal species (cow, sheep, goat and mixtures of them) isolated from Plate Count Agar.

Type of Milk	Microbial Group	Percentage (%)	Species	Percentage (%)
Cow	Lactic acid bacteria	*79.31*	*Lactococcus lactis*	48.28
			*Lactiplantibacillus plantarum*	31.03
	*Staphylococcaceae*	*17.24*	*Staphylococcus epidermidis*	10.34
			*Mammaliicoccus vitulinus*	3.45
			*Staphylococcus pasteuri*	3.45
	*Enterobacterales*	*3.45*	*Klebsiella oxytoca*	3.45
Goat	Lactic acid bacteria	79.60	*Lactococcus lactis*	38.99
			*Lacticaseibacillus paracasei*	10.17
			*Lactiplantibacillus plantarum*	8.48
			*Enterococcus durans*	11.86
			*Enterococcus faecium*	6.77
			*Enterococcus faecalis*	1.69
			*Enterococcus malodoratus*	1.69
	*Stahphyloococcaceae*	11.87	*Staphylococcus equorum*	10.18
			*Staphylococcus epidermidis*	1.69
	*Micrococcaceae*	1.69	*Micrococcus luteus*	1.69
	*Enterobacterales*	1.69	*Hafnia alvei*	1.69
	Other Gram-negative bacteria	5.09	*Psychrobacter alimentarius*	5.09
Mixture	Lactic acid bacteria	100	*Lactococcus lactis*	100
Sheep	Lactic acid bacteria	93.52	*Lactococcus lactis*	48.92
			*Leuconostoc mesenteroides*	17.02
			*Lacticaseibacillus paracasei*	9.11
			*Lactiplantibacillus plantarum*	4.56
			*Levilactobacillus brevis*	1.67
			*Pediococcus pentosaceus*	1.20
			*Leuconostoc citreum*	0.48
			*Enterococcus faecalis*	4.08
			*Enterococcus hirae*	2.88
			*Enterococcus durans*	2.16
			*Enterococcus gilvus*	1.20
			*Enterococcus faecium*	0.24
	*Staphylococcaceae*	3.60	*Staphylococcus equorum*	2.40
			*Staphylococcus hominis*	0.96
			*Staphylococcus epidermidis*	0.24
	*Enterobacterales*	0.48	*Hafnia alvei*	0.48
	Yeasts	0.24	*Debaryomyces hansenii*	0.24
	Other Gram-positive bacteria	1.68	*Bacillus cereus_thuringiensis_PG_IV*	0.48
			*Brochothrix thermosphacta*	0.48
			*Microbacterium lacticum*	0.48
			*Microbacterium paraoxydans*	0.24
	Other Gram-negative bacteria	0.48	*Acinetobacter schindleri*	0.48

**Table 4:** Lactic acid bacteria identified in cheese made from pasteurized and raw milk from different animal species (cow, sheep, goat and mixtures of them) isolated from MRS.

Milk Treatment	Milk Type	Species	Percentage (%)
Pasteurized	Cow	*Lactococcus lactis*	70.00
		*Lacticaseibacillus rhamnosus*	30.00
	Goat	*Lactococcus lactis*	69.23
		*Lacticaseibacillus paracasei*	5.13
		*Leuconostoc mesenteroides*	27.08
		*Pediococcus pentosaceus*	2.56
	Sheep	*Lacticaseibacillus paracasei*	50.00
		*Lactococcus lactis*	40.00
		*Enterococcus faecalis*	10.00
Raw	Cow	*Lacticaseibacillus paracasei*	53.34
		*Lactiplantibacillus plantarum*	40.00
		*Lactococcus lactis*	3.33
		*Leuconostoc pseudomesenteroides*	3.33
	Goat	*Lacticaseibacillus paracasei*	53.33
		*Lactiplantibacillus plantarum*	31.67
		*Lactococcus lactis*	6.67
		*Leuconostoc pseudomesenteroides*	5.00
		*Leuconostoc mesenteroides*	3.33
	Mixture	*Lactococcus lactis*	96.67
		*Lacticaseibacillus paracasei*	33.33
	Sheep	*Lacticaseibacillus paracasei*	51.71
		*Lactiplantibacillus plantarum*	11.22
		*Loigolactobacillus coryniformis*	10.49
		*Lactococcus lactis*	9.51
		*Lacticaseibacillus rhamnosus*	7.07
		*Levilactobacillus brevis*	6.59
		*Lentilactobacillus diolivorans*	0.98
		*Leuconostoc mesenteroides*	0.49
		*Pediococcus pentosaceus*	0.49
		*Latilactobacillus curvatus*	0.24
		*Lactiplantibacillus paraplantarum*	0.24
		*Latilactobacillus sakei*	0.24
		*Enterococcus faecalis*	0.49
		*Enterococcus faecium*	0.24

**Table 5:** Enterococci identified in cheese made from pasteurized (P) and raw (R) milk from different animal species (cow, sheep, goat and mixtures of them) isolated from Bile Esculin Azide agar.

Milk Treatment	Milk Type	Species	Percentage (%)
Pasteurized	Goat	*Enterococcus faecalis*	75.00
		*Enterococcus faecium*	12.50
		*Enterococcus malodoratus*	12.50
Raw	Cow	*Enterococcus durans*	62.50
		*Enterococcus malodoratus*	25.00
		*Enterococcus faecalis*	12.50
	Goat	*Enterococcus durans*	50.00
		*Enterococcus faecium*	34.67
		*Enterococcus faecalis*	15.38
	Mixture	*Enterococcus faecium*	87.50
		*Enterococcus durans*	12.50
	Sheep	*Enterococcus faecalis*	31.06
		*Enterococcus durans*	20.00
		*Enterococcus faecium*	18.95
		*Enterococcus gilvus*	16.84
		*Enterococcus hirae*	12.10
		*Enterococcus malodoratus*	1.05

**Table 6:** Staphylococci identified in cheese made from pasteurized and raw milk from different animal species (cow, sheep, goat and mixtures of them) isolated from Mannitol Salt Agar (MSA).

Milk Treatment	Milk Type	Species	Percentage (%)
Pasteurized	Cow	*Staphylococcus equorum*	100
	Goat	*Staphylococcus equorum*	100
	Sheep	*Staphylococcus equorum*	100
Raw	Cow	*Staphylococcus equorum*	82.76
		*Staphylococcus epidermidis*	17.24
	Goat	*Staphylococcus equorum*	100
	Mixture	*Staphylococcus equorum*	90.00
		*Staphylococcus capitis*	10.00
	Sheep	*Staphylococcus equorum*	69.76
		*Staphylococcus simulans*	14.63
		*Staphylococcus saprophyticus*	4.87
		*Staphylococcus pasteuri*	2.92
		*Staphylococcus epidermidis*	2.44
		*Staphylococcus ureilyticus*	1.95
		*Staphylococcus haemolyticus*	0.98
		*Mammaliicoccus sciuri*	0.49
		*Staphylococcus aureus*	0.49
		*Staphylococcus capitis*	0.49
		*Staphylococcus hominis*	0.49
		*Staphylococcus piscifermentans*	0.49

**Table 7:** *Enterobacterales* identified in cheese made from pasteurized and raw milk from different animal species (cow, sheep, goat and mixtures of them) isolated from VRBGA.

Milk Treatment	Milk Type	Species	Percentage (%)
Pasteurized	Goat	*Lelliottia amnigena*	77.78
		*Enterobacter bugandensis*	11.11
		*Enterobacter hormaechei*	11.11
Raw	Cow	*Hafnia alvei*	45.45
		*Raoultella ornithinolytica*	31.82
		*Escherichia coli*	9.09
		*Klebsiella oxytoca*	9.09
		*Raoultella planticola*	4.55
	Goat	*Hafnia alvei*	100
	Sheep	*Hafnia alvei*	90.63
		*Escherichia coli*	6.25
		*Leclercia adecarboxylata*	3.12

**Table 8:** Yeasts identified in cheese made from pasteurized and raw milk from different animal species (cow, sheep, goat and mixtures of them) isolated from PDA.

Milk Treatment	Milk Type	Species	Percentage (%)
Pasteurized	Cow	*Debaryomyces hansenii*	100
	Goat	*Debaryomyces hansenii*	83.33
		*Yarrowia lipolytica*	11.11
		*Kluyveromyces marxianus*	5.56
	Sheep	*Debaryomyces hansenii*	100
Raw	Cow	*Debaryomyces hansenii*	100
	Goat	*Debaryomyces hansenii*	47.63
		*Kluyveromyces lactis*	33.33
		*Kluyveromyces marxianus*	9.52
		*Pichia fermentans*	4.76
		*Yarrowia lipolytica*	4.76
	Sheep	*Debaryomyces hansenii*	54.84
		*Candida zeylanoides*	22.58
		*Kluyveromyces lactis*	9.68
		*Clavispora lusitaniae*	8.06
		*Yarrowia lipolytica*	4.84

## Data Availability

The data will be available upon request.

## Supplementary Materials

The following supporting information can be downloaded at: https://www.mdpi.com/article/10.3390/foods15040721/s1, Table S1. Microbial counts (log CFU/g) found in pasteurized and raw milk cheese samples. Table S2. Microbial counts (log CFU/g) found in raw milk cheese made with different types of milk (sheep, goat, cow milk or mixture of them).

## References

[1] Mayo B., Rodríguez J., Vázquez L., Flórez A.B. (2021). Microbial interactions within the cheese ecosystem and their application to improve quality and safety. Foods.

[2] Choi J., Jung Y., Kim H., Park H., Lee S. (2020). Microbial communities of a variety of cheeses and comparison between core and rind region of cheeses. J. Dairy Sci..

[3] Yang C., Zhao F., Hou Q., Wang J., Li M., Sun Z. (2020). PacBio sequencing reveals bacterial community diversity in cheeses collected from different regions. J. Dairy Sci..

[4] Banjara N., Suhr M.J., Hallen-Adams H.E. (2015). Diversity of yeast and mold species from a variety of cheese types. Curr. Microbiol..

[5] Dugat-Bony E., Garnier L., Denonfoux J., Ferreira S., Sarthou A.S., Bonnarme P., Irlinger F. (2016). Highlighting the microbial diversity of 12 French cheese varieties. Int. J. Food Microbiol..

[6] Cenci-Goga B., Cruciani D., Crotti S., Karama M., Yıldırım G., Bulut M., Marino C., Giansanti L. (2021). Diversity of yeasts and moulds in dairy products from Umbria, central Italy. J. Dairy Res..

[7] Ritschard J.S., Van Loon H., Amato L., Meile L., Schuppler M. (2022). High prevalence of *Enterobacterales* in the smear of surface-ripened cheese with contribution to organoleptic properties. Foods.

[8] Roselli M., Colafranceschi F., Cipriani V., Valle A., Zinno P., Guantario B., Schifano E., Uccelletti D., Devirgiliis C. (2025). Isolation and Characterization of Lactic Acid Bacteria from an Italian Traditional Raw Milk Cheese: Probiotic Properties and Technological Performance of Selected Strains. Microorganisms.

[9] Praça J., Furtado R., Coelho A., Correia C.B., Borges V., Gomes J.P., Pista A., Batista R. (2023). *Listeria monocytogenes*, *Escherichia coli* and coagulase positive *Staphylococci* in cured raw milk cheese from Alentejo region, Portugal. Microorganisms.

[10] Hussein N.D., Hassan J.W., Osman M., El-Omari K., Kharroubi S.A., Toufeili I., Kassem I.I. (2023). Assessment of the Microbiological Acceptability of White Cheese (Akkawi) in Lebanon and the Antimicrobial Resistance Profiles of Associated *Escherichia coli*. Antibiotics.

[11] Deddefo A., Mamo G., Asfaw M., Edao A., Hiko A., Fufa D., Jafer M., Sombo M., Amenu K. (2024). Occurrence, antimicrobial susceptibility, and resistance genes of *Staphylococcus aureus* in milk and milk products in the Arsi highlands of Ethiopia. BMC Microbiol..

[12] Bonilla-Luque O.M., Possas A., Valero A. (2020). Calidad microbiológica del queso curado artesanal elaborado con leche cruda de cabra producido en España. e-Gnosis.

[13] Imre K., Ban-Cucerzan A., Herman V., Sallam K.I., Cristina R.T., Abd-Elghany S.M., Morar D., Popa S.A., Imre M., Morar A. (2022). Occurrence, pathogenic potential and antimicrobial resistance of *Escherichia coli* isolated from raw milk cheese commercialized in Banat Region, Romania. Antibiotics.

[14] Menezes K.V., Pimentel B.M.F., Da Costa J.A.C., Ferreira N.S., Ignacchiti M.D.C., Resende J.A. (2023). Virulence factors and antimicrobial resistance of *Escherichia coli* isolated from commercialized fresh cheese in the south of Espírito Santo. Braz. J. Microbiol..

[15] Kuzeubayeva A., Ussenbayev A., Aydin A., Akanova Z., Rychshanova R., Abdullina E., Seitkamzina D., Sakharia L., Ruzmatov S. (2024). Contamination of Kazakhstan cheeses originating from *Escherichia coli* and its resistance to antimicrobial drugs. Vet. World.

[16] Ribeiro L.F., Rossi G.A.M., Sato R.A., De Souza Pollo A., Cardozo M.V., Amaral L.A.D., Fairbrother J.M. (2024). Epidemiology, virulence and antimicrobial resistance of *Escherichia coli* isolated from small Brazilian farms producers of raw milk fresh cheese. Microorganisms.

[17] Vázquez L., Sredni M.E., Rodríguez J., Flórez A.B., Mayo B. (2023). Antibiotic resistance/susceptibility profiles of *Staphylococcus equorum* strains from cheese, and genome analysis for antibiotic resistance genes. Int. J. Mol. Sci..

[18] Refaat S.S., Erdem Z.A., Kasapoğlu M.Z., Ortakcı F., Dertli E. (2025). Assessing the antibiotic resistance in food lactic acid bacteria: Risks in the era of widespread probiotic use. Food Sci. Nutr..

[19] Santamarina-García G., Amores G., Llamazares D., Hernández I., Barron L.J.R., Virto M. (2024). Phenotypic and genotypic characterization of antimicrobial resistances reveals the effect of the production chain in reducing resistant lactic acid bacteria in an artisanal raw ewe milk PDO cheese. Food Res. Int..

[20] Perez-Arnedo I., Cantalejo M.J., Martinez-Laorden A., Gonzalez-Fandos E. (2021). Effect of processing on the microbiological quality and safety of chicken carcasses at slaughterhouse. Int. J. Food Sci. Technol..

[21] da Silva-Guedes J., Martinez-Laorden A., Gonzalez-Fandos E. (2022). Effect of the presence of antibiotic residues on the microbiological quality and antimicrobial resistance in fresh goat meat. Foods.

[22] Gonzalez-Fandos E., da Silva-Guedes J. (2024). Microbiological quality and antibiotic resistance of relevant bacteria from horsemeat. Microorganisms.

[23] CLSI (2020). Performance Standards for Antimicrobial Susceptibility Testing.

[24] Quigley L., O’Sullivan O., Beresford T.P., Ross R.P., Fitzgerald G.F., Cotter P.D. (2012). High-throughput sequencing for detection of subpopulations of bacteria not previously associated with artisanal cheeses. Appl. Environ. Microbiol..

[25] Salazar J.K., Carstens C.K., Ramachandran P., Shazer A.G., Narula S.S., Reed E., Ottesen A., Suresh K.M. (2018). Metagenomics of pasteurized and unpasteurized Gouda cheese using targeted 16S rDNA sequencing. BMC Microbiol..

[26] Kamilaria E., Anagnostopoulos D.A., Papademas P., Kamilaris A., Tsaltas D. (2020). Characterizing Halloumi cheese’s bacterial communities through metagenomic analysis. LWT.

[27] Hammad A.M., Hassan H.A., Shimamoto T. (2015). Prevalence, antibiotic resistance and virulence of *Enterococcus* spp. in Egyptian fresh raw milk cheese. Food Control.

[28] Buzatto de Souza D., Pereira R.I., Endres C.M., Frazzon J., Prichula J., Guedes Frazzon A.P. (2023). Resistant enterococci isolated from raw sheep’s milk and cheeses from South region of Brazil. Ciência Rural.

[29] Martín-Platero A.M., Valdivia E., Maqueda M., Martín-Sánchez I., Martínez-Bueno M. (2008). Polyphasic approach to bacterial dynamics during the ripening of Spanish farmhouse cheese, using culture-dependent and -independent methods. Appl. Environ. Microbiol..

[30] Yunita D., Dodd C.E.R. (2018). Microbial community dynamics of a blue-veined raw milk cheese from the United Kingdom. J. Dairy Sci..

[31] Erhardt M.M., Fröder H., Oliveira W.C., Schwan R.F. (2023). Microbial diversity in artisanal cheese produced and commercialized in Vale do Taquari in southern Brazil. Braz. J. Biol..

[32] Özadam A., Özpinar H. (2016). Phenotypic determination of ESBL- and Amp-C producing Enterobacteriaceae in cheese simples. Int. J. Food Eng. Res..

[33] Fraccalvieri R., Bianco A., Difato L.M., Capozzi L., Del Sambro L., Castellana S., Donatiello A., Serrecchia L., Pace L., Farina D. (2025). Isolation and characterization of colistin-resistant *Enterobacteriaceae* from foods in two Italian regions in the South of Italy. Microorganisms.

[34] Cardinali F., Rampanti G., Paderni G., Milanović V., Ferrocino I., Reale A., Boscaino F., Raicevic N., Ilincic M., Osimani A. (2024). comprehensive study on the autochthonous microbiota, volatilome, physico-chemical, and morpho-textural features of Montenegrin Njeguški cheese. Food Res. Int..

[35] Garnier L., Valence F., Pawtowski A., Auhustsinava-Galerne L., Frotté N., Aroncelli R., Deniel F., Coton E., Mounier J. (2017). Diversity of spoilage fungi associated with various French dairy products. Int. J. Food Microbiol..

[36] Výrostková J., Regecová I., Dudriková E., Marcinčák S., Vargová M., Kováčová M., Mal’ová J. (2021). Antimicrobial Resistance of *Enterococcus* sp. Isolated from Sheep and Goat Cheeses. Foods.

[37] Sri Prabakusuma A., Zhu J., Shi Y., Ma Q., Zhao Q., Yang Z., Xu Y., Huang A. (2022). Prevalence and antimicrobial resistance profiling of *Staphylococcus aureus* isolated from traditional cheese in Yunnan, China. Biotech.

[38] Madec J.Y., Haenni M., Nordmann P., Poirel L. (2017). Extended-spectrum beta-lactamase/AmpC- and carbapenemase-producing *Enterobacteriaceae* in animals: A threat for humans?. Clin. Microbiol. Infect..

[39] Yaici L., Haenni M., Saras E., Boudehouche W., Touati A., Madec J.Y. (2016). blaNDM-5-carrying IncX3 plasmid in *Escherichia coli* ST1284 isolated from raw milk collected in a dairy farm in Algeria. J. Antimicrob. Chemother..

