# Supplementary material for: Microbiological Quality of Spanish Aged Cheeses and the Antimicrobial Resistance Profiles of Associated Enterococci, Staphylococci and Enterobacterales
Source: Foods. 2026 Feb 15;15(4):721. doi: 10.3390/foods15040721 (PMC12939858; doi:10.3390/foods15040721)
Supplement: Supplementary file 1 [file foods-15-00721-s001.zip › foods-4104095-supplementary.pdf]

Table S1. Microbial counts (log CFU/g) found in pasteurized and raw milk cheese samples

Microbial group	Milk treatment	N[1]	ND[2]	Minimum counts	Maximum counts	Mean± Standard deviation
Mesophiles	Pasteurised	6	0	5.03	8.16	6.82±1.09 [a]
	Raw	54	0	3.16	8.38	6.82±1.20 [a]
Lactic acid bacteria	Pasteurised	6	0	3.43	8.22	6.83±1.67[a]
	Raw	54	0	3.54	8.66	7.39±1.11[a]
enterococci	Pasteurised	6	3	4.01	6.05	4.94±0.84[a]
	Raw	54	8	2.24	7.23	5.24±1.33[b]
staphylococci	Pasteurised	6	0	2.99	6.01	3.94±1.01[a]
	Raw	54	24	2.30	6.53	4.01±1.03[b]
Enterobacterales	Pasteurised	6	5	3.13	3.13	3.13±0.00[a]
	Raw	54	47	1.30	6.73	3.25±1.87[a]
Yeast	Pasteurised	6	0	3.30	6.27	4.62±1.05[a]
	Raw	54	33	2.45	5.43	3.96±0.93[a]

[1] Number of samples

[2] ND, number of samples with counts below the detection limit <2 log CFU/g, except for *Enterobacterales* in which the detection limit was <1 log CFU/g

[3]Averages in the column and microbial group sharing a superscript letter show no significant differences between pasteurised and raw cheese samples (p>0.05).

Table S2. Microbial counts (log CFU/g) found in raw milk cheese made with different types of milk (sheep, goat, cow milk or mixture of them)

Microbial group	Type of milk	N[1]	ND[2]	Minimum counts	Maximum counts	Mean± Standard deviation
Mesophiles	Sheep	41	0	3.16	8.38	6.70±1.19[a]
	Goat	6	0	4.22	8.23	6.56±1.46[a]
	Cow	3	0	7.18	8.27	7.71±0.45[ab]
	Mixture	4	0	7.24	8.12	7.73±0.32[b]
Lactic acid bacteria	Sheep	41	0	3.54	8.66	7.37±1.04[a]
	Goat	6	0	3.64	8.53	6.74±1.64[a]
	Cow	3	0	7.94	8.51	8.15±0.26[a]
	Mixture	4	0	7.53	8.53	7.94±0.42[a]
enterococci	Sheep	41	5	2.24	6.91	5.04±1.25[a]
	Goat	6	1	4.45	7.23	6.08±0.92[ab]
	Cow	3	0	5.89	7.13	6.67±0.56[b]
	Mixture	4	2	2.69	6.55	4.62±1.93[c]
staphylococci	Sheep	41	21	2.30	5.58	3.82±0.83[a]
	Goat	6	1	2.80	6.53	4.41±1.53[b]
	Cow	3	0	4.65	3.63	3.38±0.26[b]
	Mixture	4	2	3.12	6.53	4.62±1.93[ac]
Enterobacterales	Sheep	41	37	1.30	4.80	2.44±1.38[a]
	Goat	6	5	6.73	6.73	6.73±0.00[bc]
	Cow	3	1	2.15	4.14	3.15±1.00[c]
	Mixture	4	4	ND	ND	ND[ab]
Yeast	Sheep	41	28	2.45	5.32	3.75±0.84[a]
	Goat	6	2	2.6	5.43	3.78±1.08[a]
	Cow	3	0	4.54	5.21	4.84±0.28[a]
	Mixture	4	3	4.91	4.91	4.91±0.00[a]

[1] Number of samples

[2] ND, number of samples with counts below the detection limit <2 log CFU/g, except for *Enterobacterales* in which the detection limit was <1 log CFU/g

[3] Averages in the column and microbial group sharing a superscript letter show no significant differences between raw cheese made with different types of milk ($p > 0.05$).
